# Theoretical chronobiology of circadian timing—gold mine or minefield?

**DOI:** 10.1038/s44323-026-00090-4

**Published:** 2026-07-21

**Authors:** Hanspeter Herzel, Solvej Lilienthal, Peter Hammerstein, Marta del Olmo

**Affiliations:** 1 Institute for Theoretical Biology, Humboldt Universität zu Berlin and Charité Universitätsmedizin, Berlin, Germany; 2 Nature Communications, Springer Nature AG & Co. KG aA, Berlin, Germany

**Keywords:** Biophysics, Evolution, Neuroscience, Physics, Systems biology

## Abstract

From noisy single cells to coupled tissues, circadian rhythms depend on interactions across multiple scales and on external time cues. Oscillator theory has played a central role in making sense of these dynamics, particularly in explaining synchronization and entrainment. Yet the same abstractions that make oscillator models powerful can also mask biologically relevant differences between systems. In this perspective, we outline where oscillator theory has been most informative, where it can mislead, and what this means for future experimental and theoretical work. While we focus here on circadian rhythms, related principles extend to other biological oscillators across timescales.

## Introduction

Chronobiology is an interdisciplinary field. Circadian rhythms emerge from molecular feedback loops, noisy single-cell dynamics, propagate through multicellular networks, and ultimately shape organismal physiology, metabolism and behavior. They do so across species in all kingdoms of life^1,2^. Making sense of this complexity requires theory, not as an optional add-on, but as a central organizing principle^3–5^.

Over the past decades, theoretical approaches in chronobiology have expanded dramatically. High-throughput transcriptomics, long-term imaging, and single-cell reporters now generate rich datasets that require advanced data analysis and bioinformatics techniques^6,7^. In parallel, kinetic models of transcription-translation feedback loops (TTFLs) have clarified how delayed negative feedback loops can generate self-sustained oscillations^8–10^. In these approaches, concepts from nonlinear dynamics and oscillator theory (limit cycles, phase response curves, entrainment, synchronization, and bifurcations) have provided a shared language to compare clocks across experimental systems and scales^3,4^.

In this Perspective, we argue that oscillator theory has been a very productive conceptual framework in chronobiology: a *gold mine*. It has helped explain why noisy single cells can synchronize robustly^11–13^, why entrainment phases depend systematically on intrinsic periods and amplitudes^14–17^, and why small period differences can translate into large behavioral phase of entrainment differences in vertebrates^4,18,19^. These insights do not depend on molecular details. Rather, they emerge from a small number of dynamical principles that apply across biological contexts beyond circadian biology.

At the same time, oscillator theory can become a *minefield* if applied uncritically. Simplifying assumptions about oscillator type, coupling strength, or phase resetting can lead to misleading interpretations of experimental data. In practice, circadian clocks are often treated as if they were all robust limit-cycle oscillators. This assumption hides much of what matters biologically, including the contributions of noise, damping, and intercellular coupling^20–22^. Whether a rhythm is weak or strong, noise-driven, or self-sustained, is not just a technical distinction: it leads to qualitatively different expectations for how clocks synchronize, how far they can be entrained, and how flexibly they respond to perturbations.

Here, we do not aim to provide a comprehensive review. Instead, we highlight selected success stories and limitations of oscillator theory in chronobiology, focusing on two central themes: (i) how weak, noisy single-cell oscillators enable robust network-level rhythms, and (ii) how intrinsic oscillator properties and zeitgeber strength, together, determine the phase of entrainment. With a small number of concepts, we focus on situations where oscillator theory has helped understand biological systems, as well as cases where it has blurred important distinctions or left key questions unresolved.

## Sloppy cells enable robust synchronization and entrainment

A long-standing question in chronobiology is how reliable and robust circadian rhythms emerge from single cells that are anything but reliable: variable and noisy. At the organismic level, circadian clocks are self-sustained, robust and precise, as demonstrated in classic experiments under constant conditions^23,24^. In contrast, oscillations recorded in isolated single cells often show strong variability and stochasticity in amplitude, period, and phase, and frequently decay in the absence of coupling or external forcing^21,25–27^. Reconciling these two observations has been a key issue in the field.

A useful way to approach this problem is to move beyond a purely molecular description and instead consider the underlying dynamical regimes of the system. Oscillator theory provides such a framework by distinguishing how rhythms are generated, maintained, and perturbed under different conditions. In this context, oscillator strength emerges as a key organizing concept. Importantly, oscillator strength is not a binary property but spans a continuum that depends on system parameters and noise levels.

At one end of this continuum, systems may exhibit damped oscillations that require noise or external input to sustain rhythmicity^19,28–30^. At the other end, strongly self-sustained limit cycles show rapid recovery after perturbations. Importantly, weak oscillatory behavior is not restricted to damped systems: limit cycles with slow amplitude relaxation or high susceptibility to perturbations can behave effectively as weak oscillators (see ref. 19 for a more complete review of these concepts). These different regimes can arise from similar underlying feedback architectures, such as delayed negative feedback, often supported by positive feedback loops^31,32^. In this context, oscillator strength, shaped by both deterministic dynamics and stochastic fluctuations, matters at least as much as molecular details when it comes to synchronization and entrainment.

A growing body of experimental and theoretical work suggests that many mammalian single-cell circadian rhythms are better described as weak or noisy oscillators rather than strong autonomous limit cycles. Westermark et al. showed that stochastic damped oscillators can accurately reproduce long-term single-cell recordings^20^. Similar conclusions emerged from analyses of phase diffusion and amplitude variability in fibroblasts and SCN neurons^12,21^. These findings challenge early TTFL-based models that assumed robust single-cell limit cycles. Importantly, in such systems, stochastic fluctuations are not merely measurement noise but an intrinsic component of the dynamics, shaping phase diffusion, amplitude variability, and even the persistence of oscillations^28,33–35^.

From a theoretical perspective, the apparent “sloppiness” of single cells (arising from both weak restoring dynamics and intrinsic stochasticity) is not a flaw but a functional feature. Networks composed of weak or damped oscillators synchronize more readily and more robustly than networks of heterogeneous strong limit cycle oscillators^11,13,36^. In contrast, networks of coupled limit cycles are prone to complex dynamics such as quasiperiodicity, toroidal oscillations, and deterministic chaos^37^. Such behaviors are rarely observed in the SCN, which instead functions as a remarkably stable and coherent oscillator network, at least at the level of experimentally accessible observables.

The SCN exemplifies how coupling can transform weak, noisy oscillators into a strong network-level oscillator. Computational studies show that even weak coupling among damped oscillators can generate stable, high-amplitude rhythms at the population level^13,38^. Experimental recordings from individual SCN neurons support this view, revealing imprecise oscillations at the single-cell level that become highly synchronized through intercellular coupling^12,27,39^.

This distinction between weak and strong oscillators also matters for entrainment. Strong oscillators typically exhibit narrow entrainment ranges and small phase response curves (PRCs), as reviewed by the classic work of Aschoff and Pohl^16^. In contrast, weak oscillators or noise-sensitive oscillators are more easily entrained and display broader entrainment ranges. Consistent with this prediction, the SCN (as a strongly coupled network) shows a narrower entrainment range than peripheral tissues such as the lung, where coupling is weaker^36^.

Together, these findings suggest a shift in perspective: robust circadian rhythms at the organismal level do not require robust oscillators at the single-cell level. Instead, noisy and/or weakly oscillatory cells may constitute an optimal “ingredient” for synchronization and entrainment through coupling. An important open challenge is to develop experimental criteria that reliably distinguish between different dynamical regimes (including damped, wealkly self-sustained and strongly self-sustained oscillators) in vivo, and to determine how oscillator type and strength varies across tissues, developmental stages, and physiological conditions.

## The phase of entrainment is governed systematically by periods, amplitudes, and zeitgeber strength

Entrainment by external zeitgebers such as light, temperature, or feeding is essential for aligning physiology and behavior with the environment^16^. Entrainment itself has been recognized for decades; however, insights from oscillator theory show that the resulting phase relationship between the oscillator and the zeitgeber is not random but constrained by the properties of the internal clock and the nature of the zeitgeber. From an evolutionary perspective, this phase relationship is one of the most relevant outputs of the circadian system^40^, as it determines when physiology and behavior occur relative to the external world.

A classical empirical observation is that organisms with shorter intrinsic periods synchronize to the zeitgeber with an earlier phase relationship (phase of entrainment) than those with longer periods^4^. Extreme human chronotypes and sleep phase disorders, such as Familial Advanced Sleep Phase Syndrome (FASPS), provide additional examples of this relationship^41^. However, comparative studies reveal that the strength of the period-phase relationship varies dramatically across species. In unicellular organisms, plants, and insects, changes in intrinsic period often translate almost linearly into changes in entrainment phase^16,42^. In contrast, in vertebrates (and particularly in humans) small period differences can result in larger phase shifts^43,44^: for example, morning larks have about 0.2 hours shorter periods, but get up about 1.5 hours earlier^44,45^.

Oscillator theory explains these differences through the distinction between weak and strong oscillators. Weak oscillators typically exhibit broad entrainment ranges for physiologically realistic zeitgeber strengths and large phase shifts in response to perturbations^46,47^. Their phase response curves are large and often show phase resetting rather than small advances and delays. As a consequence, the phase of entrainment depends only weakly on intrinsic period.

By contrast, stronger oscillators (such as the mammalian SCN at the network level) display narrow entrainment ranges and are less susceptible to large phase shifts under perturbations^16,48–50^. In this regime, small changes in intrinsic period or coupling strength can lead to steep shifts in entrainment phase^17,51^. This provides a natural explanation for the pronounced phase differences between human chronotypes despite relatively minor period variation^43,45^.

In humans, however, the relationship between intrinsic period, zeitgeber, and phase of entrainment is further complicated by feedback from behavior onto the effective zeitgeber. Sleep timing, social schedules, and light exposure patterns all modulate the phase and strength of environmental inputs that entrain the circadian system^43,44^. As a result, entrainment in humans might be better described as a semi-closed loop system, in which the internal clock not only responds to external forcing but also actively shapes the timing of that forcing through behavioral choices^52,53^. This bidirectional coupling can amplify the impact of relatively small differences in intrinsic period on observed sleep timing, since changes in phase of entrainment alter exposure to light-dark cycles and thus feed back onto subsequent entrainment dynamics.

## Arnold tongue diagrams characterize entrainment

**Fig. 1:**
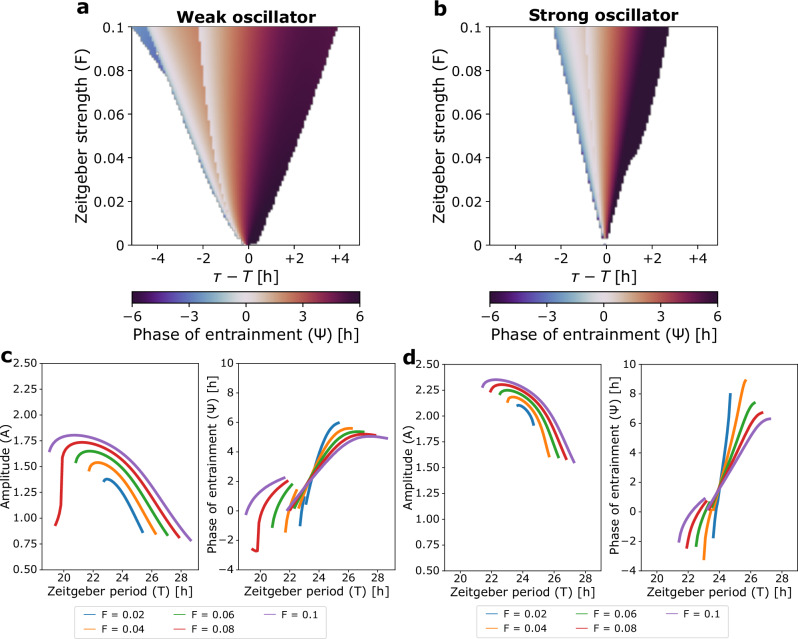
Entrainment properties of weak and strong circadian oscillators. Arnold tongue diagrams showing regions of stable 1:1 entrainment as a function of period detuning (*τ* − *T*) and zeitgeber strength (*F*) for a weak (**a**) and a strong (**b**) oscillator, computed using a Poincaré amplitude-phase model with twist^15^ ($\mathop{r}\limits^{^\circ }=\lambda {r}^{2}(A-r),\mathop{\phi }\limits^{^\circ }=\omega +\epsilon (A-r)$, where *τ* = 24 h, *λ* = 0.01 h^-1^, *ϵ* = 0.03, *A* = 1 for the weak oscillator and *A* = 2 for the strong oscillator). Color encodes the phase of entrainment (*ψ*), illustrating the broad range of stable entrainment phases and the characteristic “12-hour rule”^17^. Weak oscillators display wider entrainment ranges and shallow phase-period dependencies, whereas strong oscillators exhibit narrower entrainment ranges and steeper phase shifts. Dependence of oscillation amplitude (left) and phase of entrainment (right) on zeitgeber period *T* for increasing zeitgeber strength *F*, for weak (**c**) and strong (**d**) oscillators. Amplitude resonance peaks near zero detuning, reflecting frequency matching between oscillator and zeitgeber, while phase-period relationships differ qualitatively between weak and strong oscillators, and even show abrupt jumps.

These relationships are conveniently visualized using Arnold tongue diagrams, a central tool of oscillator theory. Arnold tongues map regions of stable frequency locking as a function of period detuning and zeitgeber strength^19,29^. Entrainment is possible if the period detuning (*τ* − *T*, where *τ* is the clock’s intrinsic period, and *T* is the zeitgeber period) is not too large and if the zeitgeber strength is sufficient. Within these regions, oscillators entrain with well-defined phases and amplitudes. Figure 1a–b illustrates entrainment behavior for weak and strong oscillators, respectively, using a Poincaré amplitude-phase model with twist.

Colors in the Arnold tongues from Fig. 1 represent the phase of entrainment *ψ*. A striking feature is the existence of a broad range of stable entrainment phases spanning approximately 12 hours, a phenomenon known as the “12-h rule”^4,17^. Oscillator theory explains this behavior through resonance arguments, phase response curves and the Kuramoto model^17,51^. Importantly, large phase variability within the entrainment range has been repeatedly observed in experimental studies across taxa^16,54,55^.

In addition to phase, entrainment affects oscillation amplitude. As shown in Figure 1c–d, amplitudes often peak near zero detuning, reflecting resonance between the intrinsic oscillator and the zeitgeber^19^. Such amplitude resonances are not just theoretical curiosities: experiments from the Johnson lab have demonstrated that resonant entrainment can accelerate growth in cyanobacteria^40^. These effects cannot be observed in purely phase-based descriptions but emerge naturally in amplitude-phase models.

Figure 1 highlights a key conceptual difference between weak and strong oscillators. For weak oscillators, wide entrainment ranges combined with the 12-hour rule result in shallow dependencies of phase on period. In contrast, strong oscillators exhibit steep phase-period relationships. This distinction provides a unifying explanation for diverse entrainment behaviors, and underscores why network coupling reshapes circadian timing. Amplitude and phase discontinuities are also evident in Fig. 1. These abrupt transitions, along with skewed resonance curves, are linked to twist^15,56^. Although such stepwise changes are rarely observed in natural circadian clocks, theoretical models predict that these transitions require a precise interplay of twist, nonlinearity, and damping.

Together, these results illustrate how oscillator theory reduces molecular and physiological complexity to a small set of dynamical parameters: intrinsic period, zeitgeber period, amplitude, zeitgeber strength, and coupling. At the same time, they highlight a potential pitfall: neglecting oscillator strength or amplitude dynamics can lead to incorrect conclusions about entrainment mechanisms. Identifying which dynamical regime applies in a given biological context remains a central challenge for future experimental and theoretical work.

## Oscillator theory as a minefield?

The success of oscillator theory in chronobiology in these last decades should not hide its limitations. Reduced dynamical models can be very informative, but they also risk oversimplification. Applying oscillator concepts without checking their assumptions can lead to misleading interpretations, especially when biological clocks are assigned to inappropriate dynamical categories.

A first major pitfall concerns the misclassification of oscillator type. Early theoretical models of TTFLs often assumed that single circadian cells behave as strong, self-sustained limit cycle oscillators with pronounced dead zones in their phase response curves^8–10^. However, quantitative analyses of long-term single-cell recordings have revealed a more nuanced picture: most mammalian single-cell rhythms are better described as weak oscillators or noise-driven damped systems^20,21^. Assuming strong-oscillator behavior in these cases can bias conclusions about synchronization, entrainment range, and phase resetting.

A second point concerns how phase response curves (PRCs) are defined and measured across different contexts. In experimental circadian biology, PRCs quantify the phase shift induced by a perturbation of finite strength and duration, such as a light pulse, and therefore depend on stimulus amplitude, timing, and protocol. In theoretical approaches, however, related quantities are often defined in the weak-perturbation limit, such as infinitesimal phase response curves (iPRCs)^57^ or velocity response curves (VRCs)^58^, which are derived from the underlying dynamical system. While these constructs are related and can coincide under specific assumptions, they correspond to different operational regimes and should not be used interchangeably.

This distinction is particularly relevant when interpreting PRCs as signatures of intrinsic oscillator properties^48^. Their shape can depend on oscillator strength, noise level, and zeitgeber intensity, in addition to experimental design. In weak or noise-dominated oscillators, apparent phase resetting may reflect transient relaxation dynamics rather than stable limit-cycle phase shifts. Furthermore, features such as slow amplitude recovery or overdrive suppression through gradual period drifts^59^ can decouple single-pulse phase shifts from entrainment behavior under periodic forcing^60^. Taken together, PRCs provide a useful but protocol-dependent characterization of oscillator response, and are best interpreted alongside measures of amplitude dynamics and entrainment stability.

A third, less explored issue concerns multistability and birhythmicity^61,62^. Nonlinear oscillator theory predicts that multiple stable rhythms can coexist under identical conditions, as seen in classical systems such as the Duffing oscillator. Although clear experimental evidence for such behavior in circadian systems is rare, ignoring this possibility can bias the interpretation of entrainment transitions, rhythm splitting, or abrupt phase shifts. In fact, phenomena such as rhythm splitting have been shown to occur under specific experimental or physiological conditions^63,64^, as well as in mathematical models of the TTFL^61,65^ (see also Fig. 1). Keeping in mind multistable regimes in theoretical analyses might be a reminder that qualitatively different solutions may exist under similar conditions.

Networks of coupled oscillators introduce additional layers of complexity. Depending on coupling strength, topology, and phase relationships, such systems can display quasiperiodicity, toroidal dynamics, or even deterministic chaos^3,37,65^. The fact that these behaviors are rarely observed in the SCN is in itself informative: it constrains the relevant dynamical regimes and supports the view that weak single-cell oscillators, combined with appropriately structured coupling, give rise to stable network rhythms^11^.

Last, biological oscillators are inherently stochastic, and that this has important consequences for how deterministic concepts should be interpreted. In particular, quantities such as oscillator strength (well-defined in deterministic systems in terms of the stability of limit cycles, basin structure, or response to perturbations) acquire a probabilistic meaning in stochastic settings, where intrinsic noise can blur attractor structure, induce switching between coexisting states, or even suppress weak oscillations altogether. As a result, regimes such as multistability, birhythmicity, or quasiperiodicity, which are sharply defined in deterministic phase space, may become difficult or effectively impossible to resolve in single-cell experimental data, not only due to measurement uncertainty but because stochastic fluctuations can qualitatively alter the long-term dynamics. This does not invalidate the deterministic framework, but it highlights that its dynamical objects should be interpreted as noise-robust organizing centers rather than directly observable states. In this sense, “oscillator strength” in biological systems is more appropriately understood as a measure of robustness of rhythmicity to stochastic perturbations, rather than strict dynamical stability in the deterministic sense. Recognizing this distinction is essential when using deterministic phase space structures as interpretative tools for inherently stochastic biological oscillators.

Recognizing these *minefields* does not weaken the value of oscillator theory. Instead, it clarifies how and when the framework can be used. When applied in an informative manner to oscillator type, noise, and coupling regimes, reduced models remain a powerful way to organize and interpret oscillatory data. The challenge for future work is not to abandon reduced models, but to use them carefully, guided by quantitative data and explicit tests of the underlying assumptions.

## Key insights and open challenges in theoretical chronobiology

To conclude, we highlight key insights and remaining challenges in theoretical chronobiology using a compact Q&A format. This approach is not meant to be exhaustive, but rather to illustrate where oscillator theory has provided clear guidance, and where careful interpretation is still required.

**Q1: How are self-sustained circadian oscillations generated?**

Self-sustained oscillations arise from nonlinear feedback systems, most commonly through delayed negative feedback loops and network switches. In circadian clocks, the effective delay typically amounts to roughly a quarter of the period, corresponding to about 6 h^66,67^. While positive feedback is not strictly required, it can stabilize oscillations and enlarge parameter regimes that support rhythmicity^31,32^. Importantly, the existence of a feedback loop alone does not guarantee a strong limit cycle: oscillator strength depends on kinetic parameters, noise levels, and coupling (see ref. ^19^ for a comprehensive review).

**Q2: Are single cells self-sustained circadian oscillators?**

In some unicellular organisms, yes. Certain cyanobacteria exhibit remarkably stable, self-sustained oscillations over months^68^. By contrast, multicellular organisms exhibit a different pattern. In many mammalian cell types, for example, single-cell rhythms are weak, noisy, or even damped, yet persist through stochastic forcing^20,21^. Treating all single cells as strong limit cycle oscillators hides the dynamic diversity and can lead to incorrect predictions about synchronization and entrainment^36^. Weak oscillators, while imprecise in isolation, can be highly functional in networks.

**Q3: How can coupling between circadian oscillators be quantified?**

Coupling in circadian systems arises through diverse biochemical, mechanical, and electrical mechanisms. In the SCN, neuropeptide signaling or gap junctions contribute to coupling^39,69^; in fibroblasts and hepatocytes, paracrine signaling or mechanical contact has been suggested to couple networks of cells^22,70–73^. Quantifying coupling strength directly is challenging, but theory suggests several practical indicators. Weak coupling manifests as partial frequency pulling and broad phase distributions, whereas stronger coupling leads to phase locking, amplitude enhancement, and resonance effects^38^. The phase of coupling (not just its magnitude) can critically determine synchronization outcomes^74–76^.

**Q4: How do collective circadian properties emerge from single-cell dynamics?**

Collective circadian rhythms arise from interactions between heterogeneous and often noisy cellular oscillators. Depending on coupling strength, heterogeneity, and external inputs, these systems can exhibit a range of collective behaviors, including full synchronization, phase-locked states with non-zero phase differences, or partially coherent and misaligned regimes^38,77^. Thus, synchronization represents only one possible outcome of collective dynamics. Coupling, nonlinearity, and timescale separation determine whether population-level behavior reflects simple averaging or qualitatively new emergent properties, such as enhanced robustness, coherence, or flexibility in response to perturbations. Understanding which dynamical regime is realized in a given biological system remains an open challenge.

**Q5: How can the complexity of coupled oscillator systems be visualized?**

Despite their biological complexity, coupled oscillator systems can often be reduced to a small number of control parameters, most notably period mismatch and coupling or zeitgeber strength. Arnold tongue diagrams provide a compact visualization of frequency locking, entrainment ranges, and transitions to quasiperiodic or chaotic dynamics^3,19,29^. Such representations help identify biologically plausible regimes and exclude others that, while mathematically possible, are unlikely to occur in vivo.

**Q6: How can fast and stable entrainment be achieved?**

Weak oscillators are generally easier to entrain than strong ones, particularly under physiologically realistic zeitgeber strengths^36,47^. Theoretical studies indicate that appropriately timed combinations of zeitgebers (such as light and feeding) can substantially accelerate entrainment^78–80^. These insights suggest that entrainment speed is not solely a property of the clock, but can be optimized through external control strategies.

**Q7: What determines the phase of entrainment?**

The phase of entrainment is governed jointly by intrinsic oscillator properties (period, amplitude, oscillator strength) and zeitgeber characteristics (strength, waveform, photoperiod)^19,51^. Oscillator theory predicts systematic dependencies that differ qualitatively between weak and strong oscillators. In particular, strong oscillators exhibit steep phase-period relationships and shallow resonance curves, whereas weak oscillators follow broader phase rules and broader resonance effects (Fig. 1). Ignoring oscillator strength can therefore lead to misinterpretation of phase data across species and tissues.

**Q8: What can oscillator theory contribute to medical applications?**

Medical applications such as cancer chronotherapy involve multiple interacting oscillatory processes, including circadian clocks, cell-cycle dynamics, and drug pharmacokinetics^81–84^. Oscillator theory offers a framework for identifying optimal treatment times and schedules that maximize efficacy while minimizing side effects^19,85–87^. However, successful translation requires realistic assumptions about oscillator coupling and variability, underscoring the need for theory-driven experimental validation.

**Q9: Is noise always detrimental to circadian oscillations?**

Noise is often viewed as a source of variability that degrades precision by introducing variability in rhythmic parameters. However, in circadian systems, intrinsic stochasticity can play a more nuanced role. In weak or near-critical systems oscillators, noise can sustain oscillations, shape phase diffusion, and facilitate transitions between dynamical regimes^28,33–35^. At the population level, noise can even enhance synchronization by enabling flexible adjustment to coupling and external cues. At the same time, excessive noise can disrupt coherence and reduce robustness. Understanding how noise interacts with oscillator strength, coupling, and external forcing remains an important challenge for both theory and experiment.

**Q10: What experimental advances are needed to inform theory?**

A central challenge is that many dynamical quantities inferred in theoretical models are not directly measured in current experiments. Progress will require experimental designs that go beyond passive observation. First, systematically varying zeitgeber strength, waveform, and period would allow reconstruction of entrainment maps and Arnold tongues, providing direct constraints on oscillator strength and nonlinear response properties. Second, long-term single-cell recordings combined with transient perturbations (e.g., pulses or step changes) are needed to quantify amplitude relaxation rates, phase diffusion, and recovery dynamics, which distinguish weak, noise-driven, and self-sustained oscillators. Third, controlled perturbations of intercellular coupling (through genetic, pharmacological, or optogenetic approaches) would enable quantitative inference of coupling strength and phase relationships in oscillator networks. Finally, experiments designed to probe hysteresis, multistability, or noise-induced switching (e.g., through slow parameter sweeps or repeated perturbations) would provide critical tests of nonlinear dynamical predictions. Such datasets, combining single-cell resolution with controlled inputs, are essential to calibrate and validate both deterministic and stochastic models.

## Concluding remarks

Together, these questions highlight both the power and the limits of oscillator theory in chronobiology. Reduced dynamical models capture complex molecular and cellular processes into a small set of intuitive parameters, providing guidance on synchronization, entrainment, and phase regulation across scales. At the same time, careful attention to oscillator type, noise, coupling, and attention to multistability is essential to avoid overinterpretation. Going forward, combining quantitative experiments with theory-driven modeling will be critical to understand how circadian clocks function across tissues, respond to environmental changes, and impact physiology and health. With this approach, theoretical chronobiology can continue to reveal general principles and help guide experimental studies.

## Data Availability

No datasets were generated or analysed during the current study.

## References

[1] Dunlap, J. C. & Loros, J. J. Making time: conservation of biological clocks from fungi to animals. *Microbiol. Spectr.* **5**, 10–1128 (2017). 10.1128/microbiolspec.funk-0039-2016 PMC5446046 28527179

[2] Rijo-Ferreira, F. & Takahashi, J. S. Genomics of circadian rhythms in health and disease. *Genome Med.* **11**, 82 (2019). 31847894 10.1186/s13073-019-0704-0 PMC6916512

[3] Winfree, A. T. *The Geometry of Biological Time* Vol. 2 (Springer, 1980).

[4] Wever, R. Zum Mechanismus der biologischen 24-Stunden-Periodik. *Kybernetik* **1**, 139–154 (1962). 14320326 10.1007/BF00306797

[5] Kronauer, R. E., Czeisler, C. A., Pilato, S. F., Moore-Ede, M. C. & Weitzman, E. D. Mathematical model of the human circadian system with two interacting oscillators. *Am. J. Physiol.* **242**, R3–R17 (1982). 7058927 10.1152/ajpregu.1982.242.1.R3

[6] Hughes, M. E. et al. Harmonics of circadian gene transcription in mammals. *PLoS Genet* **5**, e1000442 (2009). 19343201 10.1371/journal.pgen.1000442 PMC2654964

[7] Talamanca, L., Gobet, C. & Naef, F. Sex-dimorphic and age-dependent organization of 24-hour gene expression rhythms in humans. *Science* **379**, 478–483 (2023). 36730411 10.1126/science.add0846

[8] Becker-Weimann, S., Wolf, J. H. H. & Kramer, A. Modeling feedback loops of the mammalian circadian oscillator. *Biophys. J.* **87**, 3023–34 (2004). 15347590 10.1529/biophysj.104.040824 PMC1304775

[9] Leloup, J.-C. & Goldbeter, A. Toward a detailed computational model for the mammalian circadian clock. *Proc. Natl. Acad. Sci. USA* **100**, 7051–7056 (2003). 12775757 10.1073/pnas.1132112100 PMC165828

[10] Forger, D. B. & Peskin, C. S. A detailed predictive model of the mammalian circadian clock. *Proc. Natl. Acad. Sci. USA* **100**, 14806–14811 (2003). 14657377 10.1073/pnas.2036281100 PMC299807

[11] Gonze, D., Bernard, S., Waltermann, C., Kramer, A. & Herzel, H. Spontaneous synchronization of coupled circadian oscillators. *Biophys. J.* **89**, 120–129 (2005). 15849258 10.1529/biophysj.104.058388 PMC1366510

[12] Webb, A. B., Angelo, N., Huettner, J. E. & Herzog, E. D. Intrinsic, nondeterministic circadian rhythm generation in identified mammalian neurons. *Proc. Natl. Acad. Sci. USA* **106**, 16493–16498 (2009). 19805326 10.1073/pnas.0902768106 PMC2752526

[13] Bernard, S., Gonze, D., Čajavec, B., Herzel, H. & Kramer, A. Synchronization-induced rhythmicity of circadian oscillators in the suprachiasmatic nucleus. *PLoS Comp. Biol.* **3**, e68 (2007). 10.1371/journal.pcbi.0030068 PMC1851983 17432930

[14] Schmal, C., Myung, J., Herzel, H. & Bordyugov, G. A theoretical study on seasonality. *Front Neurol.* **6**, 94 (2015). 25999912 10.3389/fneur.2015.00094 PMC4423511

[15] Del Olmo, M. et al. Are circadian amplitudes and periods correlated? A new twist in the story. *F1000Res* **12**, 1077 (2024). 37771612 10.12688/f1000research.135533.2 PMC10526121

[16] Aschoff, J. & Pohl, H. Phase relations between a circadian rhythm and its zeitgeber within the range of entrainment. *Naturwissenschaften* **65**, 80–84 (1978). 345129 10.1007/BF00440545

[17] Granada, A. E., Bordyugov, G., Kramer, A. & Herzel, H. Human chronotypes from a theoretical perspective. *PLoS One* **8**, e59464 (2013). 23544070 10.1371/journal.pone.0059464 PMC3609763

[18] Roenneberg, T., Hut, R., Daan, S. & Merrow, M. Entrainment concepts revisited. *J. Biol. Rhythms* **25**, 329–339 (2010). 20876813 10.1177/0748730410379082

[19] Del Olmo, M., Ector, C. & Herzel, H. Time after time—circadian clocks through the lens of oscillator theory. *FEBS Lett.* **600**, 808–836 (2026). 41566861 10.1002/1873-3468.70257 PMC13022753

[20] Westermark, P. O., Welsh, D. K., Okamura, H. & Herzel, H. Quantification of circadian rhythms in single cells. *PLoS Comp. Biol.* **5**, e1000580 (2009). 10.1371/journal.pcbi.1000580 PMC2776301 19956762

[21] Leise, T. L., Wang, C. W., Gitis, P. J. & Welsh, D. K. Persistent cell-autonomous circadian oscillations in fibroblasts revealed by six-week single-cell imaging of PER2::LUC bioluminescence. *PLoS One* **7**, e33334 (2012). 22479387 10.1371/journal.pone.0033334 PMC3315561

[22] Finger, A.-M. et al. Intercellular coupling between peripheral circadian oscillators by TGF-*β* signaling. *Sci. Adv.* **7**, eabg5174 (2021). 34301601 10.1126/sciadv.abg5174 PMC8302137

[23] D’Ortous de Mairan, J.-J. Observation botanique. *Histoire de l’Académie Royale des Sciences Paris*. **31**, 35–36 (1729).

[24] Aschoff, J. Circadian rhythms in man: a self-sustained oscillator with an inherent frequency underlies human 24-hour periodicity. *Science* **148**, 1427–1432 (1965). 14294139 10.1126/science.148.3676.1427

[25] Michel, S., Geusz, M. E., Zaritsky, J. J. & Block, G. D. Circadian rhythm in membrane conductance expressed in isolated neurons. *Science* **259**, 239–241 (1993). 8421785 10.1126/science.8421785

[26] Nagoshi, E. et al. Circadian gene expression in individual fibroblasts: cell-autonomous and self-sustained oscillators pass time to daughter cells. *Cell* **119**, 693–705 (2004). 15550250 10.1016/j.cell.2004.11.015

[27] Yamaguchi, S. et al. Synchronization of cellular clocks in the suprachiasmatic nucleus. *Science* **302**, 1408–1412 (2003). 14631044 10.1126/science.1089287

[28] Gu, C., Xu, J., Rohling, J., Yang, H. & Liu, Z. Noise induces oscillation and synchronization of the circadian neurons. *PLoS One* **10**, e0145360 (2015). 26691765 10.1371/journal.pone.0145360 PMC4687094

[29] Balanov, A., Janson, N., Postnov, D. & Sosnovtseva, O. *Synchronization: From Simple to Complex* (Springer, Heidelberg, 2009).

[30] Dani, C. et al. Seasonal cycles select for self-sustained circadian oscillators. *Curr. Biol.* **36**, 1541–1547 (2026). 10.1016/j.cub.2026.01.012 PMC12880617 41638208

[31] Ananthasubramaniam, B. & Herzel, H. Positive feedback promotes oscillations in negative feedback loops. *PLoS One* **9**, e104761 (2014). 25126951 10.1371/journal.pone.0104761 PMC4134231

[32] Goldbeter, A. et al. *Biochemical Oscillations And Cellular Rhythms* (Cambridge University Press, 1997).

[33] Purcell, O., Savery, N. J., Grierson, C. S. & Di Bernardo, M. A comparative analysis of synthetic genetic oscillators. *J. R. Soc. Interface* **7**, 1503–1524 (2010). 20591848 10.1098/rsif.2010.0183 PMC2988261

[34] Hou, Z. & Xin, H. Internal noise stochastic resonance in a circadian clock system. *J. Chem. Phys.* **119**, 11508–11512 (2003).

[35] Zou, X., Wang, K. & Fan, D. Stochastic Poincaré–Bendixson theorem and its application on stochastic Hopf bifurcation. *Int J. Bifurc. Chaos* **23**, 1350070 (2013).

[36] Abraham, U. et al. Coupling governs entrainment range of circadian clocks. *Mol. Syst. Biol.* **6**, 438 (2010). 21119632 10.1038/msb.2010.92 PMC3010105

[37] Linsay, P. S. & Cumming, A. W. Three-frequency quasiperiodicity, phase locking, and the onset of chaos. *Phys. D.* **40**, 196–217 (1989).

[38] Schmal, C., Herzog, E. D. & Herzel, H. Measuring relative coupling strength in circadian systems. *J. Biol. Rhythms* **33**, 84–98 (2018). 29219034 10.1177/0748730417740467 PMC6344889

[39] Herzog, E. D., Hermanstyne, T., Smyllie, N. J. & Hastings, M. H. Regulating the suprachiasmatic nucleus (SCN) circadian clockwork: interplay between cell-autonomous and circuit-level mechanisms. *Cold Spring Harb. Perspect. Biol.* **9**, a027706 (2017). 28049647 10.1101/cshperspect.a027706 PMC5204321

[40] Ouyang, Y., Andersson, C. R., Kondo, T., Golden, S. S. & Johnson, C. H. Resonating circadian clocks enhance fitness in cyanobacteria. *Proc. Natl. Acad. Sci. USA* **95**, 8660–8664 (1998). 9671734 10.1073/pnas.95.15.8660 PMC21132

[41] Xu, Y. et al. Modeling of a human circadian mutation yields insights into clock regulation by PER2. *Cell* **128**, 59–70 (2007). 17218255 10.1016/j.cell.2006.11.043 PMC1828903

[42] Merrow, M., Brunner, M. & Roenneberg, T. Assignment of circadian function for the Neurospora clock gene frequency. *Nature* **399**, 584–586 (1999). 10376598 10.1038/21190

[43] Roenneberg, T., Wirz-Justice, A. & Merrow, M. Life between clocks: daily temporal patterns of human chronotypes. *J. Biol. Rhythms* **18**, 80–90 (2003). 12568247 10.1177/0748730402239679

[44] Duffy, J. F., Rimmer, D. W. & Czeisler, C. A. Association of intrinsic circadian period with morningness–eveningness, usual wake time, and circadian phase. *Behav. Neurosci.* **115**, 895 (2001). 11508728 10.1037//0735-7044.115.4.895

[45] Duffy, J. F. & Wright Jr, K. P. Entrainment of the human circadian system by light. *J. Biol. Rhythms* **20**, 326–338 (2005). 16077152 10.1177/0748730405277983

[46] Daan, S. & Pittendrigh, C. S. A functional analysis of circadian pacemakers in nocturnal rodents. II. The variability of phase response curves. *J. Comp. Physiol.* **106**, 253–266 (1976).

[47] Granada, A. E. & Herzel, H. How to achieve fast entrainment? The timescale to synchronization. *PloS One* **4**, e7057 (2009). 19774087 10.1371/journal.pone.0007057 PMC2745570

[48] Johnson, C. H. Phase response curves: what can they tell us about circadian clocks. *Circadian Clocks from Cell to Human* 209–249 (1992).

[49] Comas, M., Beersma, D., Spoelstra, K. & Daan, S. Phase and period responses of the circadian system of mice (*Mus musculus*) to light stimuli of different duration. *J. Biol. Rhythms* **21**, 362–372 (2006). 16998156 10.1177/0748730406292446

[50] Khalsa, S. B. S., Jewett, M. E., Cajochen, C. & Czeisler, C. A. A phase response curve to single bright light pulses in human subjects. *J. Physiol.* **549**, 945–952 (2003). 12717008 10.1113/jphysiol.2003.040477 PMC2342968

[51] Bordyugov, G. et al. Tuning the phase of circadian entrainment. *J. R. Soc. Interface* **12**, 20150282 (2015). 26136227 10.1098/rsif.2015.0282 PMC4528595

[52] Mistlberger, R. E. & Skene, D. J. Nonphotic entrainment in humans? *J. Biol. Rhythms* **20**, 339–352 (2005). 16077153 10.1177/0748730405277982

[53] Stothard, E. R. et al. Circadian entrainment to the natural light-dark cycle across seasons and the weekend. *Curr. Biol.* **27**, 508–513 (2017). 28162893 10.1016/j.cub.2016.12.041 PMC5335920

[54] Hoffmann, K. Synchronisation der circadianen Aktivitätsperiodik von Eidechsen durch Temperaturcyclen verschiedener Amplitude. *Z. f.ür. Vgl. Physiol.* **58**, 225–228 (1968).

[55] Pittendrigh, C. S. & Daan, S. A functional analysis of circadian pacemakers in nocturnal rodents: I. The stability and lability of spontaneous frequency. *J. Comp. Physiol.* **106**, 223–252 (1976).

[56] Duffing, G. *Erzwungene Schwingungen bei veränderlicher Eigenfrequenz und ihre technische Bedeutung* 41–42 (Vieweg, 1918).

[57] Granada, A., Hennig, R. M., Ronacher, B., Kramer, A. & Herzel, H. Phase response curves: elucidating the dynamics of coupled oscillators. *Methods Enzymol.* **454**, 1–27 (2009). 19216921 10.1016/S0076-6879(08)03801-9

[58] Taylor, S. R., Webb, A. B., Smith, K. S., Petzold, L. R. & Doyle III, F. J. Velocity response curves support the role of continuous entrainment in circadian clocks. *J. Biol. Rhythms* **25**, 138–149 (2010). 20348465 10.1177/0748730409360949

[59] Kunysz, A., Glass, L. & Shrier, A. Overdrive suppression of spontaneously beating chick heart cell aggregates: experiment and theory. *Am. J. Physiol.* **269**, H1153–H1164 (1995). 7573512 10.1152/ajpheart.1995.269.3.H1153

[60] Strogatz, S. H. Interpreting the human phase response curve to multiple bright-light exposures. *J. Biol. Rhythms* **5**, 169–174 (1990). 2133126 10.1177/074873049000500208

[61] Leloup, J.-C. & Goldbeter, A. Chaos and birhythmicity in a model for circadian oscillations of the per and tim proteins in Drosophila. *J. Theor. Biol.* **198**, 445–459 (1999). 10366496 10.1006/jtbi.1999.0924

[62] Del Olmo, M. et al. Network switches and their role in circadian clocks. *J. Biol. Chem.* **300**, 107220 (2024). 38522517 10.1016/j.jbc.2024.107220 PMC11044057

[63] Daan, S. & Berde, C. Two coupled oscillators: simulations of the circadian pacemaker in mammalian activity rhythms. *J. Theor. Biol.* **70**, 297–313 (1978). 633922 10.1016/0022-5193(78)90378-8

[64] Granada, A. E., Cambras, T., Díez-Noguera, A. & Herzel, H. Circadian desynchronization. *Interface Focus* **1**, 153–166 (2011). 22419981 10.1098/rsfs.2010.0002 PMC3262243

[65] Van Soest, I., Del Olmo, M., Schmal, C. & Herzel, H. Nonlinear phenomena in models of the circadian clock. *J. R. Soc. Interface* **17**, 20200556 (2020). 32993432 10.1098/rsif.2020.0556 PMC7536064

[66] Novák, B. & Tyson, J. J. Design principles of biochemical oscillators. *Nat. Rev. Mol. Cell Biol.* **9**, 981–991 (2008). 18971947 10.1038/nrm2530 PMC2796343

[67] MacDonald, N. *Time Lags in Biological Models* Vol. 27 (Springer Science & Business Media, 2013).

[68] Mihalcescu, I., Hsing, W. & Leibler, S. Resilient circadian oscillator revealed in individual cyanobacteria. *Nature* **430**, 81–85 (2004). 15229601 10.1038/nature02533

[69] Aton, S. J. & Herzog, E. D. Come together, right… now: synchronization of rhythms in a mammalian circadian clock. *Neuron* **48**, 531–534 (2005). 16301169 10.1016/j.neuron.2005.11.001 PMC1780025

[70] Noguchi, T., Wang, L. L. & Welsh, D. K. Fibroblast PER2 circadian rhythmicity depends on cell density. *J. Biol. Rhythms* **28**, 183–192 (2013). 23735497 10.1177/0748730413487494 PMC3760388

[71] Sinturel, F. et al. Circadian hepatocyte clocks keep synchrony in the absence of a master pacemaker in the suprachiasmatic nucleus or other extrahepatic clocks. *Genes Dev.* **35**, 329–334 (2021). 33602874 10.1101/gad.346460.120 PMC7919413

[72] Abenza, J. F. et al. Mechanical control of the mammalian circadian clock via YAP/TAZ and TEAD. *J. Cell Biol.* **222**, e202209120 (2023). 37378613 10.1083/jcb.202209120 PMC10308087

[73] Guenthner, C. J. et al. Circadian rhythms of Per2::Luc in individual primary mouse hepatocytes and cultures. *PloS One* **9**, e87573 (2014). 24498336 10.1371/journal.pone.0087573 PMC3911982

[74] Morelli, L. G. et al. Delayed coupling theory of vertebrate segmentation. *HFSP J.* **3**, 55–66 (2009). 19492022 10.2976/1.3027088 PMC2689616

[75] Ananthasubramaniam, B., Herzog, E. D. & Herzel, H. Timing of neuropeptide coupling determines synchrony and entrainment in the mammalian circadian clock. *PLoS Comp. Biol.* **10**, e1003565 (2014). 10.1371/journal.pcbi.1003565 PMC3990482 24743470

[76] Tokuda, I. T., Ono, D., Honma, S., Honma, K.-I. & Herzel, H. Coherency of circadian rhythms in the SCN is governed by the interplay of two coupling factors. *PLoS Comp. Biol.* **14**, e1006607 (2018). 10.1371/journal.pcbi.1006607 PMC6301697 30532130

[77] Zavala, E. Misaligned hormonal rhythmicity: mechanisms of origin and their clinical significance. *J. Neuroendocrinol.* **34**, e13144 (2022). 35514212 10.1111/jne.13144 PMC9286602

[78] Diekman, C. O. & Bose, A. Entrainment maps: a new tool for understanding properties of circadian oscillator models. *J. Biol. Rhythms* **31**, 598–616 (2016). 27754956 10.1177/0748730416662965

[79] Grabe, S., Mahammadov, E., Olmo, M. D. & Herzel, H. Synergies of multiple zeitgebers tune entrainment. *Front Netw. Physiol.* **1**, 803011 (2022). 36925578 10.3389/fnetp.2021.803011 PMC10013031

[80] Serkh, K. & Forger, D. B. Optimal schedules of light exposure for rapidly correcting circadian misalignment. *PLoS Comp. Biol.* **10**, e1003523 (2014). 10.1371/journal.pcbi.1003523 PMC3983044 24722195

[81] Bernard, S., Čajavec Bernard, B., Lévi, F. & Herzel, H. Tumor growth rate determines the timing of optimal chronomodulated treatment schedules. *PLoS Comp. Biol.* **6**, e1000712 (2010). 10.1371/journal.pcbi.1000712 PMC2841621 20333244

[82] Gutu, N. et al. Circadian coupling orchestrates cell growth. *Nat. Phys.* **21**, 768–777 (2025).

[83] Gérard, C. & Goldbeter, A. Entrainment of the mammalian cell cycle by the circadian clock: modeling two coupled cellular rhythms. *PLoS Comp. Biol.* **8**, e1002516 (2012). 10.1371/journal.pcbi.1002516 PMC3364934 22693436

[84] Droin, C., Paquet, E. R. & Naef, F. Low-dimensional dynamics of two coupled biological oscillators. *Nat. Phys.* **15**, 1086–1094 (2019). 32528550 10.1038/s41567-019-0598-1 PMC7289635

[85] Lévi, F. A. et al. Chronomodulated versus fixed-infusion-rate delivery of ambulatory chemotherapy with oxaliplatin, fluorouracil, and folinic acid (leucovorin) in patients with colorectal cancer metastases: a randomized multi-institutional trial. *J. Natl. Cancer Inst.* **86**, 1608–1617 (1994). 7932825 10.1093/jnci/86.21.1608

[86] Ector, C. et al. Time-of-day effects of cancer drugs revealed by high-throughput deep phenotyping. *Nat. Commun.* **15**, 7205 (2024). 39169017 10.1038/s41467-024-51611-3 PMC11339390

[87] Wang, C. et al. Circadian tumor infiltration and function of CD8+ T cells dictate immunotherapy efficacy. *Cell* **187**, 2690–2702 (2024). 38723627 10.1016/j.cell.2024.04.015

